# Complete coding sequence of influenza A virus subtype H1N3 from guinea fowl in Republic of Serbia

**DOI:** 10.1128/mra.00213-26

**Published:** 2026-07-29

**Authors:** Dejan Vidanović, Jelena Maletić, Milanko Šekler, Bojana Tešović, Nikola Vasković, Marko Dmitrić, Biljana Đurđević, Edoardo Giussani, Gianpiero Zamperin

**Affiliations:** 1 Veterinary Specialized Institute Kraljevo, Kraljevo, Serbia; 2 Institute of Veterinary Medicine of Serbia, Belgrade, Serbia; 3 Scientific Veterinary Institute "Novi Sad", Novi Sad, Serbia; 4 Viral Genomics and Transcriptomics Laboratory, Department for Research and Innovation, Istituto Zooprofilattico Sperimentale delle Venezie 83372, Legnaro, Italy; Katholieke Universiteit Leuven, Leuven, Belgium

**Keywords:** influenza A virus, H1N3, whole-genome sequencing, guinea fowl, avian viruses

## Abstract

Here, we report the complete coding sequence of the avian influenza A virus subtype H1N3 from guinea fowl [A/Guinea fowl/Serbia/Vinca_household/2025(H1N3)] obtained on the Oxford Nanopore Technologies MinION device in combination with an amplicon-based protocol.

## ANNOUNCEMENT

Influenza in birds is caused by infection with viruses from the genus *Alphainfluenzavirus*, family *Orthomyxoviridae*. Avian influenza virus (AIV) can be classified into 16 HA and 9 NA subtypes, with certain strains posing a serious threat to animal health and public health due to their zoonotic potential (1, 2). Continuous monitoring of AIV is essential for early detection of emerging strains, timely outbreak response, and effective implementation of control measures (3–5). Whole-genome sequencing plays a crucial role in this process, as it enables detailed characterization of viral genetic changes, identification of mutations associated with increased virulence or host adaptation, and tracing of transmission pathways across regions (6–8).

During routine testing of cloacal swabs from guinea fowl (*Numida meleagris*), as part of the National Avian Influenza Monitoring Program, in a household located in a suburban settlement in Vinca (geolocation 44.756111, 20.618611) on 24 April 2025, the presence of AIV was detected by the Scientific Veterinary Institute of Serbia. For complete characterization, the sample was sent to the National Reference Laboratory (NRL) for avian influenza and Newcastle disease virus at the Veterinary Specialized Institute Kraljevo (VSIKV). The virus tested negative for the H5 (9) and H7 (10) subtypes, and virus isolation in embryonated eggs (11) was unsuccessful. For identification and typing purposes, the complete viral genome was sequenced on an Oxford Nanopore Technologies MinION (ONT, UK) device following ONT protocol “Influenza virus sequencing from RNA using SQK-NBD114” (V INF_9189_v114_revN_07Oct2025). Viral RNA was extracted from cloacal swabs of guinea fowl using the RNeasy Mini Kit (Qiagen, Germany), and genome amplification was performed using the SuperScript III Platinum One-Step qRT-PCR Kit (Invitrogen, USA). The sequencing library was prepared using the Native Barcoding Kit 24 V14 (SQK-NBD114.24, Oxford Nanopore Technologies, UK), and sequencing was performed with an FLO114 flow cell on a MinION device (Oxford Nanopore Technologies, UK) with a minimum quality of 9. Raw POD5 files were basecalled using DORADO v.1.1.1 (12) with standard parameters. In total, 1,591,679 raw reads were obtained (N50 – 1,206, GC content 44.6%), with an average length of 830 (range 5–16.101).

The consensus sequence was generated using MEDAKA v.1.1.3 (13) with standard parameters and sequences EPI2072029 (PB2), EPI2175886 (PB1), EPI1813341 (PA), EPI1657059 (HA), EPI2073614 (NP), EPI1774562 (NA), EPI1581277 (MP), and EPI1774308 (NS) as reference. Briefly, MEDAKA is a tool to create consensus sequences from nanopore sequencing data by using neural networks applied to a pileup of individual sequencing reads against a reference sequence. About 3.4% of MinION-generated reads resulted in alignment to the reference sequence by Minimap2 during the MEDAKA mapping internal step. In Table 1, the average coverage for each segment and the closest related strain are reported.

**TABLE 1:** Number of mapping reads and average coverage obtained for each AIV segment and the most closely related strain

Segment	# Mapping reads	Coverage	Accession number of the closest GenBank hit	Name of the strain most closely related	Nucleotide sequence similarity to the closest GenBank hit
PB1	274	43	OP135949.1	A/duck/Chernogolovka/5908/2021(H3N8)	98.7
PB2	214	35	PQ755265.1	A/Mallard Duck/Republic of Georgia/2/2018(H10N7)	98.0
PA	251	65	PQ756152.1	A/Mallard Duck/Republic of Georgia/6/2018(H3N8)	98.2
HA	968	369	PQ756378.1	A/Mallard Duck/Republic of Georgia/18/2018(H1N5)	98.2
NP	4,245	1,874	PQ756384.1	A/Mallard Duck/Republic of Georgia/18/2018(H1N5)	98.5
NA	4,755	1,594	MT407083.1	A/Anas platyrhynchos/Belgium/8862_H170636/2016(H5N3)	98.1
MP	19,444	2,498	PP754250.1	A/duck/Moscow/6147/2022(H3N8)	98.8
NS	24,268	2,306	OP135955.1	A/duck/Chernogolovka/5908/2021(H3N8)	99.6

This study describes a case of infection of guinea fowl with influenza A virus subtype H1N3. Although the H1N3 subtype avian influenza virus is classified as low pathogenic, its circulation in bird populations represents a potential genetic reservoir for reassortment with highly pathogenic avian influenza strains, which may lead to the emergence of new variants with altered biological properties in birds and in mammals (14). The avian influenza H1N3 genome generated in this study provides a valuable resource for future studies and improves our understanding of the genetic changes associated with the circulation, evolution, and potential emergence of new strains of both low- and highly pathogenic avian influenza viruses in natural environments (15, 16).

## Data Availability

Raw reads generated in this study have been deposited in the Sequence Read Archive database under the accession number PRJNA1402895. The genome of AIV generated here can be assessed in GenBank under accession numbers HA PZ017553, MP PZ017554, NA PZ017555, NP PZ017556, NS PZ017557, PA PZ017558, PB PZ017559, PB2 PZ017560.
